# PennCNV in whole-genome sequencing data

**DOI:** 10.1186/s12859-017-1802-x

**Published:** 2017-10-03

**Authors:** Leandro de Araújo Lima, Kai Wang

**Affiliations:** 1 Zilkha Neurogenetic Institute, University of Southern California, Los Angeles, 90089 CA USA; 2 Present address: Gladstone Institute of Neurological Disease, J. Gladstone Institutes, 1650 Owens St, San Francisco, 94158 CA USA; 3 Present address: Institute for Genomic Medicine, Columbia University, New York, 10032 USA; 4 Department of Biomedical Informatics, Columbia University, New York, 10032 USA

**Keywords:** Copy-number variation, Whole-genome sequencing, PennCNV

## Abstract

**Background:**

The use of high-throughput sequencing data has improved the results of genomic analysis due to the resolution of mapping algorithms. Although several tools for copy-number variation calling in whole genome sequencing have been published, the noisy nature of sequencing data is still a limitation for accuracy and concordance among such tools. To assess the performance of PennCNV original algorithm for array data in whole genome sequencing data, we processed mapping (BAM) files to extract coverage, representing log R ratio (LRR) of signal intensity, and B allele frequency (BAF).

**Results:**

We used high quality sample NA12878 from the recently reported NIST database and created 10 artificial samples with several CNVs spread along all chromosomes. We compared PennCNV-Seq with other tools with general deletions and duplications, as well as for different number of copies and copy-neutral loss-of-heterozygosity (LOH).

**Conclusion:**

PennCNV-Seq was able to find correct CNVs and can be integrated in existing CNV calling pipelines to report accurately the number of copies in specific genomic regions.

## Background

Several tools have been published to call copy-number variants (CNVs) in whole genome data, but the accuracy of results still remains a challenge [1]. Besides that, most of current tools do not provide the option to distinguish heterozygous calls, inherited exclusively from either mother or father, from homozygous calls, inherited from both parents simultaneously. Furthermore, to our knowledge, there are no tools to identify copy-neutral loss-of-heterozygosity (LOH) events, which are regions in the genome with two copies inherited only from one parent, and consequently have all SNPs with only one allele.

PennCNV [2] has been successfully used in array data to call CNVs since its publication in 2007. Because of its performance, it has been applied in numerous genetic studies [3–7]. The precise hidden Markov model (HMM) algorithm has delivered CNV calls that have been correctly validated biologically in most CNV studies. However, in last years, the number of sequencing studies increased and the number of samples available with high-throughput sequencing methods is large, for both whole genome and exome data.

To assess the performance of PennCNV in whole genome data, we adapted sequencing data to extract the same information available in array data, naming this new method PennCNV-Seq. We used real sample with validated CNV calls and created 10 artificial samples with different types of CNVs spread in all chromosomes. We used the well-studied 1000 genomes sample NA12878, which was recently massively sequenced by different methods and analyzed by different labs [8]. For the simulated samples, we used a tool developed by our lab (SVGen, available at https://github.com/WGLab/SVGen/) after we were not able to find simulation tools to combine artificial single-nucleotide variant (SNVs) and indels with artificial structural variations (SVs), reporting the breakpoint coordinates correctly.

We tested the performance of PennCNV-Seq with one real sample with 30X of coverage and 10 artificial samples with 20X of coverage, each artificial sample with 10 CNVs per chromosome. The results showed that PennCNV-Seq is comparable to existing tools and its validation step can be added to existing pipelines together with other tools to make reliable CNV calls.

## Methods

### Pre-processing of mapping (BAM) files

The first step can be executed in parallel for each chromosome, for each sample. From the BAM file, which is the file with sequences aligned to a chosen reference, the script “convert_map2signal.pl” generates two measures: sequence count, which will simulate log R ratio (LRR) from array data and B allele frequency (BAF), measures used by original PennCNV [2] from array chips. The program SAMtools [9] is used to calculate the coverage (with mpileup) and call the variants (with bcftools). Sequence count refers to the normalized sequence read (coverage) on either a SNV or as the average coverage in a continuous segment of genomic positions without SNVs. For this step, it is required as input the mapping (BAM) file and the reference genome (FASTA file).

#### Choice of SNP markers

Array chips were originally created for genome-wide association studies using SNPs, and the markers are chosen taking common variants in the population. PennCNV-Seq uses a combination of SNPs common in population and the SNPs present in the sample. To increase the accuracy, regions between pairs of SNPs are also used as markers and contribute to the algorithm with coverage (LRR) information. As the resulting “B allele frequency” data has to be compared to the expected allele frequency values in the population, which is taken from 1000 genomes project [10] and downloaded from ANNOVAR [11] database. There are different files for each of these super populations (sets of populations): ALL (all samples), AFR (African), AMR (Ad Mixed American), EAS (East Asian), EUR (European) and SAS (South Asian). The allele frequency file can be changed for each sample being analyzed. More details can be found on the website (http://www.1000genomes.org/category/population/). So an additional step is executed to match the markers (SNPs) and regions found in the previous step with the markers present in the allele frequency data. We then used BEDTools [12] to split the previous regions in smaller regions depending on whether there are SNPs/markers from the general population inside these regions. Based on the user’s choice, this data can be downloaded for versions hg19 and hg38 of the reference genome.

#### Log R ratio

**Fig. 1:**
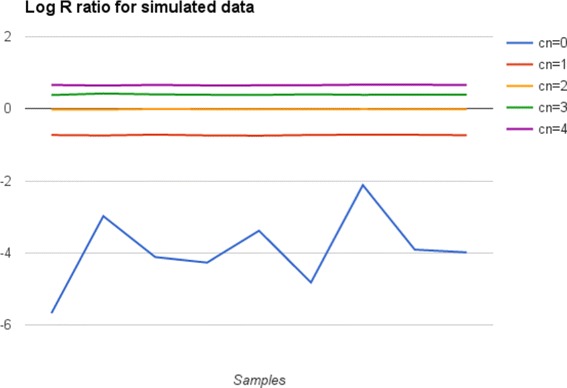
Log R ratio for simulated data, in different types of CNVs. These values are used as input for PennCNV-Seq algorithm, and were estimated for sequencing data. We generated 10 samples with 240 CNVs each, with copy-number (cn) 0, 1, 2, 3 and 4. After that, the mean LRR was generated for each region

The log R ratio (LRR) is the normalized measure of signal intensity for each SNP marker, in array chips. It is calculated taking the log2 of the ratio between the observed and expected signal for two copies of the genome. After the normalization, we expect to see the signal clustered around 0 when the region has two copies. Higher values may indicate a duplication event and lower values could be an evidence of deletion (shown as an example in Fig. 1). PennCNV-Seq extracts this value for each region taking the pileup output given by SAMtools [9]. In sequencing data, the expected coverage is calculated as the mean coverage for the corresponding chromosome. LRR of a marker or region is then calculated as the log2 of the coverage from this region divided by the mean coverage for the chromosome.

#### B allele frequency

B allele frequency (BAF) simply refers to the fraction of reads supporting non-reference alleles at a given SNV position. This measure can be extracted from aligned alleles at each position with SNV call and helps to define CNV regions. For example, in one-copy deletions, one would expect to see decreased sequence count and general lack of clustering of B allele frequency around 0.5, compared to neighboring regions without deletions. As in the original algorithm, PennCNV-Seq also uses “2” for markers without information about allele frequency.

#### Hidden Markov model (HMM)

The original algorithm of PennCNV [2] was not changed, but we tuned some parameters to work with sequencing data. Because of different patterns of sequencing data, the expected LRR had to be recalculated and used as input parameter for the HMM. Besides average, the standard deviation of coverage was calculated for regions with copy-number (CN) equals to 0, 1, 2, 3 and 4. We tested different parameters for the transition matrix and increased the probability of changing the state, that is, we decreased the probability of a state stay the same in 0.05 (from approx. 0.94 to 0.89) for CN=0, 1, 3 and 4. The probabilities for CN=2 to stay the same are still 0.999.

### Validation

#### Simulated data

To assess the performance of PennCNV in sequencing data, we generated 10 artificial samples with SNVs and 5 types of CNVs spread randomly in all chromosomes. For each sample, we generated two copies of genomes using different frequency profiles for SNVs. The first copies, simulating mother’s genomes, received SNVs in the frequency of European population (EUR code in the 1000 Genomes Project), and the second copies, simulating the father’s genomes, received SNVs with the frequency of African population (AFR code in the 1000 Genomes Project). After that, CNVs were inserted according to the following descriptions and quantities: homozygous and heterozygous deletions, respectively zero-copy (approx. 54 per sample) and one-copy CNVs (approx. 64 per sample), heterozygous and homozygous duplications, respectively three-copy (approx. 74 per sample) and four-copy CNVs (approx. 44 per sample), and loss-of-heterozygosity (approx. 4 per sample), which are two copies inherited only from mother or only from father, hence with all SNPs being homozygous. The samples were created with SVGen tool (available at https://github.com/WGLab/SVGen/), each one with average 20X of coverage. To examine the impact of SV length in the simulation, CNVs were created with lengths 1 kb, 1.5 kb, 2 kb, 2.5 kb, 3 kb, 3.5 kb, 4 kb, 5 kb, 6 kb, 8 kb, 10 kb, 20 kb, 30 kb, 40 kb, 50 kb, 75 kb, 100 kb, 150 kb, 200 kb, 500 kb, 1 mb and 5 mb, and average distance between CNV regions was 100 kb. LOH regions were simulated only with size of 5 mb. All simulated data were generated based on hg38 genome reference assembly. The next step was to generate paired-end reads with length of 100 bp and average insert size of 300 bp. Then, the reads were mapped to the original reference genome using BWA [13].

#### Real data

To analyze the performance of PennCNV-Seq in real data we used the 1000 genomes well-studied sample NA12878. In a paper published recently, Zook et al. [8] provide a series of high quality data for benchmarking of variant calling algorithms. The sample NA12878 is available from different laboratories and techniques. We used in this work the Illumina whole genome sequencing data initially with 300X of coverage downsampled to 30X (see reference for more details; data available at ftp://ftp-trace.ncbi.nlm.nih.gov/giab/ftp/data/NA12878/NIST_NA12878_HG001_HiSeq_300x). The set of 2676 deletions was used as ground truth regions.

#### Comparison with other CNV calling tools

To compare the performance of PennCNV-Seq with other tools, we used CNVnator [14], which uses read coverage to call CNVs (deletions and duplications); and Lumpy [15], which uses read-pair, split-read and read-depth; PennCNV-Seq uses read-depth and allele frequency to make CNV calls. We compared the performance of different tools considering zero- and one-copy CNVs as one single set called “deletions”, three- and four-copy CNVs as one single set called “duplications”. We did not compare the performance of calling different number of copies and loss-of-heterozygosity because available tools do not have this options. These features were tested only in PennCNV-Seq.

#### Precision and recall calculation of CNV calls

Two performance measures were calculated considering at least 70% of overlap between predicted CNVs and the real CNV call. For the ROC curve, we considered as threshold (minimal length of CNVs) for ground truth and calls CNV regions with 0 kb, 1 kb, 2 kb,..., and 50 kb.

#### Individual validation of known calls

PennCNV [2] has an option to validate the call of a given region. This step returns the likelihood of the region regarding five different HMM states, representing zero-copy, one-copy, two-copy, three-copy and four-copy regions. We applied this step in all intervals of real CNVs to check the whether the validation of real CNVs would return correct results.

## Results

Using the mapping (BAM) files as input, the pre-processing step generated approx. 4.83 markers per 1000 bases in each chromosome, which defines an approximate resolution for PennCNV calls. After this, we use the position of SNPs common in population to split the markers with large intervals in smaller regions neighbouring the common SNP positions, for each sample. This step generates BAFLRR files with millions of lines (e.g. approx. 12 millions for chrom. 1 and 2 millions for chroms. 21 and 22, but only approx. 200,000 for chrom. Y), which will increase the resolution of PennCNV-Seq.

### LRR parameters for HMM

To adapt the HMM parameters for PennCNV-Seq, we used simulated data to calculate the expected value for LRR in regions of copy-number (CN) equals to 0, 1, 2, 3 and 4. We generated 10 samples with 240 CNVs each. After that, we calculated the mean and standard deviation (sd) of LRR in CNV (0, 1, 3 and 4 copies), as well as in non-CNV regions (2 copies). The results for LRR mean and sd are: for CN=0 mean is -3.739099 (st.dev. = 2.56), for CN=1 mean is -0.727964 (sd=0.3), for CN=2 mean is 0.000000 (sd=0.16), for CN=3 mean is 0.395454 (sd=0.127), for CN=4 mean is 0.658622 (sd=0.124). More details about these values can be seen in Fig. 1.

### Validation

After creating artificial genomes in FASTA files for 10 samples with 240 CNVs each, these files were used to generate reads along all the genome, with the amount of reads changing according to GC-content. Each sample was created with average 20X coverage, with paired-end reads. Three tools were used to call CNVs in real and simulated data: PennCNV-Seq, CNVnator [14] and Lumpy [15].

#### Comparison of simple deletions and duplications in simulated data

**Fig. 2:**
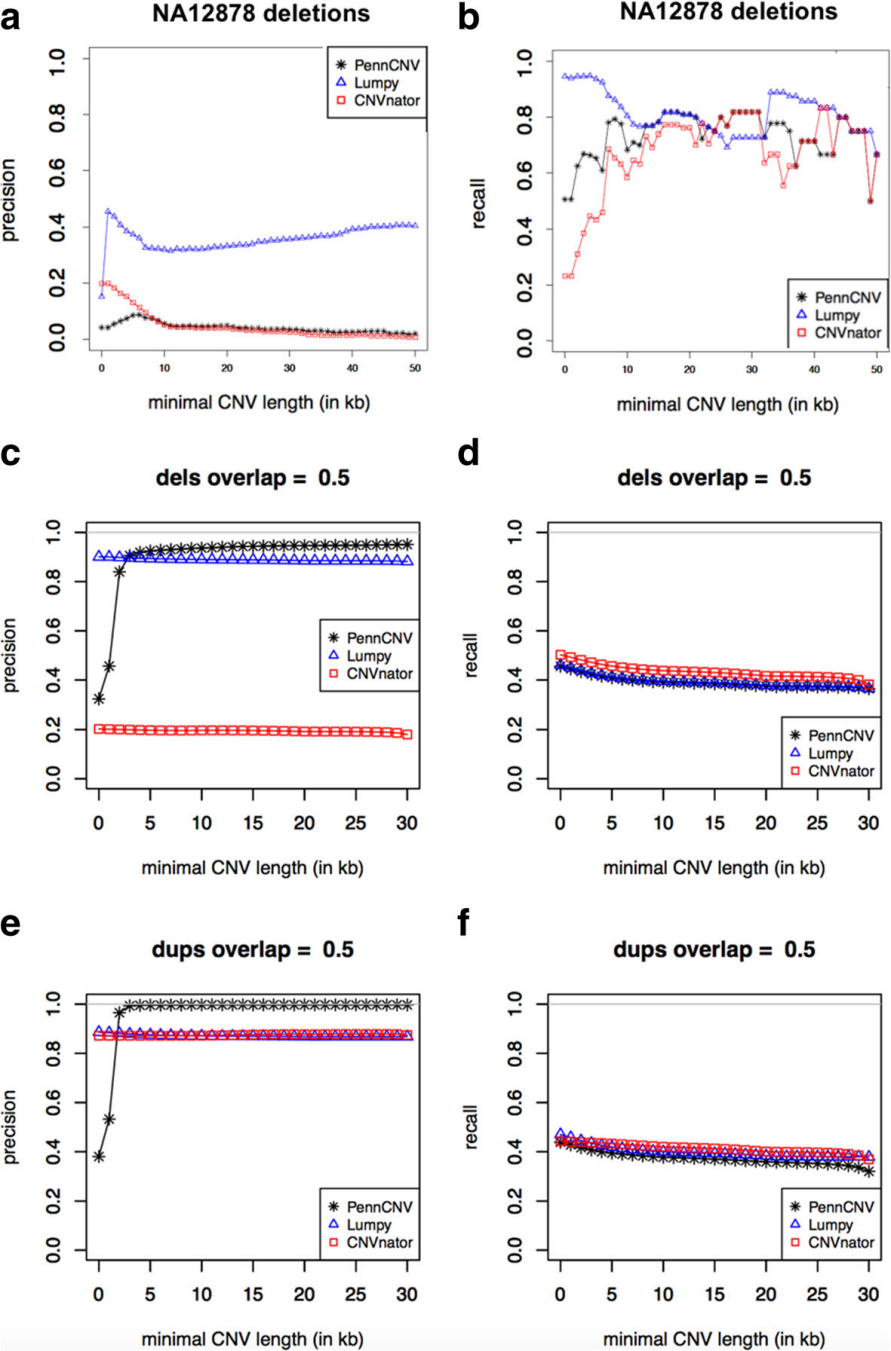
Comparison between Precision and Recall of PennCNV, Lumpy and CNVnator. **a-b** Real data: deletions of sample NA12878, with 30X coverage, downloaded from NIST project database. No duplications were reported for this sample. **c-f** Simulated data of 10 samples with 20X. c-d are showing deletions and e-f are showing duplications. The overlap to consider the prediction and the real CNV the same has to be 50%

To compare simple deletions and duplications of PennCNV-Seq with other tools, we grouped zero- and one-copy CNVs in a set that was called “deletions” and three- and four-copy CNVs in a set that was called “duplications”. We then calculated the precision and recall for each type of CNV separately and compared to the ground truth generated by SVGen (https://github.com/WGLab/SVGen/), the CNV simulator. The results are shown in Fig. 2.

#### CNV calling with different number of copies and LOH

We also tested PennCNV-Seq to assess its performance when used to detect CNVs with different number of copies: zero copy (homozygous deletion), one copy (heterozygous deletion), three copies (heterozygous duplication) or four copies (homozygous duplication). We also simulated LOH events and used the data as input for PennCNV-Seq. After that, we calculated precision and recall for each CNV type. The detailed results are shown in Table 1.

**Table 1:** Performance of PennCNV-Seq regarding different number of copies for CNVs: deletions with 0 or 1 copy, and duplication with 3 or 4 copies, and loss-of-heterozygosity (LOH)

No. of copies	Precision	Recall
0 copy (hom. deletion)	0.814	0.399
1 copy (het. deletion)	0.711	0.665
3 copy (het. duplication)	0.962	0.528
4 copy (hom. duplication)	0.732	0.416
Loss-of-heterozygosity (LOH)	1.000	0.650

Precision is TP/(TP+FP) and Recall is TP/(TP+FN), where TP=True Positive, FP=False Positive and FN=False Negative

### Calls in real data

After downloading the BAM file of NA12878, we ran PennCNV-Seq, CNVnator [14] and Lumpy [15] to find the 2675 deletions reported by NIST research [8]. We calculated recall and precision varying the threshold for minimal length of CNVs for calls and ground truth with 0 kb, 1 kb, 2 kb,..., and 50 kb. The detailed results are shown in Fig. 2.

### PennCNV’s validation step of a priori known CNV regions

**Fig. 3:**
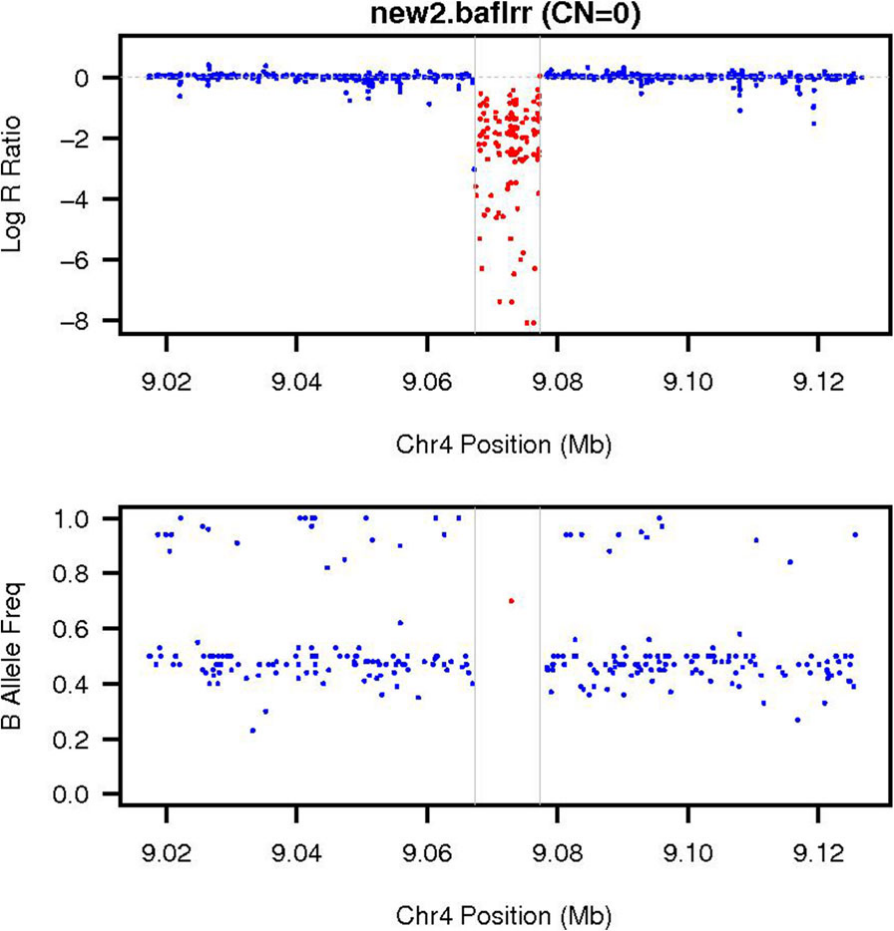
PennCNV plot of Log R Ratio (coverage) and B Allele Frequency for a zero-copy (CN=0) deletion in simulated data. It is possible to see how the coverage is much lower than the average and the lack of data for allele frequency, as there are just very few reads mapped in the read

Although PennCNV-Seq algorithm can miss some calls without prior knowledge, the validation step could be used integrated to other tools to find the correct number of copies and state of a genomic region. To check how PennCNV-Seq works to assess known CNV regions, we applied PennCNV’s validation step to ground truth regions and checked the likelihood reported for each interval. We checked visually a set of plots and compared the likelihoods to original simulation. One example can be seen in Fig. 3.

## Discussion

In last decade, the number of high-throughput sequence samples produced greatly increased. This type of data has been shown to be useful for not only short variant identification, as single-nucleotide variants (SNVs) and indels, but also for larger variants, as copy-number variations (CNVs). Several tools and methods have been published to find such types of variations in whole genome sequencing data [1, 14, 15]. Through a computational approach, each read generated by DNA sequencing machines are mapped to a reference genome, and such process generates a mapping (BAM) file with detailed information about how the short sequences match to the corresponding human genome assembly version.

Copy-number variation calling algorithms can use one or more techniques to find deletions and duplications in a genome: (i) read-pair, which compares the distance between first and second reads in the mapping to the expected insert size generated by paired-end sequencing; (ii) split-read, which extracts information from reads partially mapped to the reference, representing CNV breakpoint regions; (iii) read depth, which is the count of reads mapped to a specific region to the genome; and (iv) assembly, which uses the reads to recover the original genome and then find the CNV regions. Some tools use a combination of these four methods to improve the CNV calls. More details can be found in the recent review of Pirooznia and colleagues [1].

Although the amount of tools for CNV calling in genome data is large, the accuracy of results still remains a challenge [1]. Besides that, most of current tools do not provide the option to identify the inheritance, and consequently, distinguish the number of copies of each CNV. For example, when a tool reports a deletion or duplication, the results do not provide information about zygosity. Therefore it is not possible to know whether the CNV was inherited only from one parent or both, and this could have a big difference regarding the effect of the variation in the person.

Another important event that current CNV calling tools do not find is the copy-neutral loss-of-heterozygosity (LOH). This happens when in a specific region of a person’s genome receives two copies from the same parent, instead of receiving one copy from each parent. Thus this portion of the genome will be completely homozygous. As no other tools check information from the SNP alleles, and as in such event there is no change in number of copies, during the process of CNV calling it is not possible to identify the parts of genome in which LOH happens.

## Conclusion

With pre-processing steps to extract from mapping (BAM) files information about coverage, simulating log R ratio, and B allele frequency of SNP markers, PennCNV-Seq was able to make calls of CNVs identifying correctly zero-copy and one-copy deletions, three-copy and four-copy duplications, as well as LOH events. We were able to test PennCNV-Seq using real and simulated data, comparing the performance with existing CNV calling tools. To make the simulation more realistic, different types of variations such as SNPs, indels, deletions, and duplications are present in the simulated data. Also, GC-content bias was added to the artificial reads. However, more tests with simulated and other types of real data should be necessary to tune the input parameters of coverage mean and standard deviation for PennCNV-Seq, as the program uses this information as prior knowledge to make calls. The perfect scenario would be to have different types of validate calls with distinct number of copies available for sequencing data, but this is still a limitation.

Besides being able to generate CNV calls without a priori knowledge, PennCNV-Seq is useful to validate calls and find the zygosity of calls made by other tools. Therefore, PennCNV-Seq can be combined with other tools and integrated into existing pipelines. It is also important to emphasize that PennCNV-Seq did not commit any mistake in LOH calls for sequencing data.

## Abbreviations

BAF: B allele frequency
CNV: Copy-number variation
LOH: Loss-of-heterozygosity
LRR: Log R ratio
